# Supplementary material for: The Systems Biology Research Tool: evolvable open-source software
Source: BMC Syst Biol. 2008 Jun 29;2:55. doi: 10.1186/1752-0509-2-55 (PMC2446383; doi:10.1186/1752-0509-2-55)
Supplement: Additional file 1 — SBRT Archive. An archive of the current version of the Systems Biology Research Tool. [file 1752-0509-2-55-S1.zip › sbrt-1.4.0/doc/developers_guide/api/sbrt/kernel/fba/comp/FluxomeReducer.html]

FluxomeReducer


|  |  |  |
| --- | --- | --- |
| **Overview** \| **Package** \| **Class** \| **Use** \| **Tree** \| **Deprecated** \| **Index** \| **Help** | |  |
| **PREV CLASS**   **NEXT CLASS** | **FRAMES**    **NO FRAMES**     **All Classes** |  |
| SUMMARY: NESTED \| FIELD \| CONSTR \| METHOD | DETAIL: FIELD \| CONSTR \| METHOD |  |


---


## sbrt.kernel.fba.comp Interface FluxomeReducer<T extends Fluxome>

**Type Parameters:**: `T` - a type of fluxome.

**All Known Implementing Classes:**: MsFluxomeReducer, WwFluxomeReducer

---

``` public interface FluxomeReducer<T extends Fluxome> ```

This interface is used to represent algorithms that reduce the size of fluxomes.

**Author:**
:   This interface was written and documented by Jeremiah Wright while
    in the Wagner lab.

---

| **Method Summary** | |
| --- | --- |
| `ProgressMonitor` | `getProgress()`             Returns a progress monitor for this fluxome reducer. |
| `T` | `makeFluxome(T fluxome)`             Returns a fluxome that has been reduced in size. |

**Method Detail**

### makeFluxome

```
T makeFluxome(T fluxome)
```

:   Returns a fluxome that has been reduced in size.

    :   **Parameters:**: `fluxome` - the fluxome for which size reduction will be performed. **Returns:**: a fluxome that is smaller in size then the one provided.

---


### getProgress

```
ProgressMonitor getProgress()
```

:   Returns a progress monitor for this fluxome reducer.

    :   **Returns:**: a progress monitor for this fluxome reducer.


---


|  |  |  |
| --- | --- | --- |
| **Overview** \| **Package** \| **Class** \| **Use** \| **Tree** \| **Deprecated** \| **Index** \| **Help** | |  |
| **PREV CLASS**   **NEXT CLASS** | **FRAMES**    **NO FRAMES**     **All Classes** |  |
| SUMMARY: NESTED \| FIELD \| CONSTR \| METHOD | DETAIL: FIELD \| CONSTR \| METHOD |  |


---
